# 

**Published:** 1889-02-23

**Authors:** 


**Notice to Advertisers and Subscribers.**

All Advertisements, Orders for Copies of The Hospital, and Business Communications should be addressed to The Publisher, *not to the Editor*, The Hospital, 38, Old Jewry, E.C.

**To Residents, Secretaries, and Matrons.**

The Editor will be glad to have the Regulations and each Report as issued by Asylums, Hospitals, Convalescent and Nursing Institutions, as well as those of other Charities, and an early copy in duplicate of all Appeals and Festival Notices, etc., addressed to *The Editor, The Lodge, Porchester Square, London, W.* Any superintendent, secretary, matron, or other chief official who regularly sends such information may be registered, on application to the Publisher, to receive free of cost a cover for binding The Hospital in half-yearly volumes.

A Scale of Charges will be found on the first page of the Nursing Supplement.

### THE STUDENT'S COLUMN.

### UNIVERSITY OF LONDON.

### PRELIMINARY SCIENTIFIC (M.B.) EXAMINATION, JANUARY, 1889.

EXAMINERS:

*Chemistry:* Prof. J. Emerson Reynolds, M.D., F.R.S., and Prof. W. A. Tilden, D.Sc., F.R.S.

*Experimental Physics:* Prof. G. F. FitzGerald, M.A., F.R.S., and R. T. Glazebrook, Esq., M.A., F.R.S.

*Biology:* Prof F. Orpen Bower, D.Sc., M.A., Prof. E. Ray Lankester, M.A., F.R.S., Adam Sedgwick, Esq., M.A., F.R.S., and Prof. H. Marshall Ward, M.A., F.R.S.

PASS LIST.

ENTIRE EXAMINATION.

FIRST DIVISION.

Baker, Edwin Galliers, B.A., Owens Coll. and private study and tuition.
Davies, Hugh, Univ. Coll., Bangor, Guy's, and Charterhouse Science Sc.
Dexter, Thomas F. G., B.A., Univ. Corresp. Classes and private study.
Every-Clayton, Leopold E. V., Guy's Hospital and Birkbeck Institution.
Herring, William Conyers, St. Bartholomew's Hospital.
Poynton, Frederic John, Univ. Coll., Bristol, and St. Mary's Hospital.
Rhodes, Courtenay Mansel, Epsom College and St. Mary's Hospital.
Travers, Frederick Thomas, Blundell's School and University College.

SECOND DIVISION.

Brundrett, Gilbert Thomas, Owen's College.
Clarke, Thomas Henry, London Hospital and private study.
Gaman, Francis Robert Snuggs, Epsom and University Colleges.
Grant-Wilson, Charles W., St. Thomas's Hospital and Univ. Cor. Classes.
Henderson, Robert, Guy's Hospital and private study.
LePelley, Amelia Maitland, Ladies' C. Cheltenham, Univ. C., and priv. st.
Lowe, Lockhart, Mason College, Birmingham.
Piper, Francis Parris, Epsom College and St. Mary's Hospital.
Thompson, Herbert Campbell, Epsom College and Middlesex Hospital.

TWO SUBJECTS OF THE EXAMINATION.*

†Atkinson, J. Arthur (C., B.), St. Mary's Hospital.
Coaker, Francis W. J. (C., P.), Monmouth Gr. Sch. and London Hospital.
Cole, George (C., P.), King's College.
Eldred, Henry Wylie (C., P.), King's School, Rochester.
Gossage, Wm. Herbert (C., P.), Westminster Hospital.
Harrison, Thomas (C. P.), Easingwold Gr. School and Yorkshire College
Lyle, Herbert W. (C. B.), King's College.
Martin, Louis Charles (C., P.), R. Coll. Mauritius and University Coll.
Miskin, Ernest (C., P.), King's Col., Birkbeck Inst., and St. Thomas's Hos.
Nuttall, Arthur Peel (C., B.), Owens College and private study.
†O'Hea, Jas. Patrick (P., B.), Univ. C. Cardiff and St. Bartholomew's H.
Roberts, Walter Ll. (C., P.), Univ., St. Mary's Hosp., and private study.
Salt, Thomas (C., P.), King Edward High School, Birmingham.
Wilson, Arthur Edward (C., P.), private study.

ONE SUBJECT OF THE EXAMINATION.*

Bamfield, Harold Jn. K. (C.), King's School, Rochester, and St. Mary's.
†Cooper, Hugh Erskine (C.), St. Bartholomew's Hospital.
†Ducat, Arthur David (P.), Islington High School.

* The subjects taken up by these candidates are indicated by initials after the name: C. = Chemistry; P. = Physics; B. = Biology.
† These candidates have now completed the examination.

†Escombe, William (B.), Science Schools, Kensington, and private study.
†Flint, Thomas Buxton (P.), Owens College.
Griffiths, Arthur David (P.), Wycliffe Coll., Stonehouse, and private st.
Haigh, Thomas Frederick (C.), University College and School.
Hamilton, Dyddgu (C.), Bedford College, London.
†Harwood, Elias Francis (B.), University College and private study.
Hunter, Clement Harris (P.), Private study.
Jones, Edward Thomas (B.), Mason's College, Birmingham.
†Kingsford, Bertm. Harold (P.), St. Thomas's Hosp. and Birkbeck Inst.
†Kirby, William Egmont (C.), University College.
Knox, Robert George (C.), Dulwich and King's Colleges.
†Lloyd, Walter Hamilton (B.), Owens College.
†Moore, Hugh Myddelton (C.), St. Thomas's Hospital.
†Nicholson, John (P.), Mason and Yorkshire Colleges.
†Oldfield, Carlton (B.), Yorkshire College and Almondby Gram. School.
Parry, Leonard Arthur (C.), Guy's Hospital.
Price, William Matthews (C.), University College, Cardiff.
Rock, Frank Ernest (P.), Portsmouth Gr. S. and Middlesex Hospital.
†Rogers, Kenneth (P.), St. Bartholomew's Hospital.
†Shears, William (P.), St. Bartholomew's Hospital.
Smith, Herbert Sidney (P.), Owens College.
Thornton, Jeremiah (P.), Private study.
†Woodfield, Thomas Harold (C.), St. Bartholomew's Hospital.
Woolley, Thomas Fisher (B.), Mason College, Birmingham.

### INTERMEDIATE EXAMINATION IN MEDICINE, JANUARY, 1889.

EXAMINERS.

*Anatomy:* Prof. John Curnow, M.D., and Prof. Alex. Macalister, M.D., M.A., F.R.S.

*Physiology:* J. Newport Langley, Esq., M A., F.R.S., and Prof. E. A. Schäfer, F.R.S.

*Materia Medica, etc.:* J. Mitchell Bruce, Esq., M.D., M.A., and Frederick Taylor, Esq., M.D.

*Organic Chemistry:* Prof. J. Emerson Reynolds, M.D., F.R.S., and Prof. W. A. Tilden, D.Sc., F.R.S.

PASS LIST.

ENTIRE EXAMINATION.

FIRST DIVISION.

Caley, Henry Albert, St. Mary's Hospital.
Cooke, George Harry, Owens College and Manchester Royal Infirmary.
Hepburn, Malcolm Langton, St. Bartholomew's Hospital.
Moore, Joseph, St. Bartholomew's Hospital.
Perry, Sidney Herbert, Queen's College, Birmingham.
Pisani, Orestes Victoriano, King's College.
Rogers, William Gusterson, Guy's Hospital.

SECOND DIVISION.

Curtis, Henry Jones, University College.
Green, Douglas Richard, University College.
Hewitson, John George, University College.
Jewell, John William Frank, Guy's Hospital.
Low, Vincent Warren, St. Mary's Hospital.
Lowsley, Lionel Dewe, St. Bartholomew's Hospital.
Manknell, Arthur, the Yorkshire College.
Rows, Richard Gundry, University College.
Sheppard, Amy, London School of Medicine for Women.
Smith, Ernest Newlyn, University College.
Stevens, Thomas George, Guy's Hospital.
Thomas, John Llewellyn, St. Bartholomew's Hospital.
Umney, William Francis, St. Thomas's Hospital.
Waring, Holburt Jacob, B.Sc., St. Bartholomew's Hospital.

EXCLUDING PHYSIOLOGY.

FIRST DIVISION.

Brown, John James, Guy's Hospital.

SECOND DIVISION.

Buckley, Joseph Richard, Owens College.
James, Rupert, University College.
March, Geoffrey Colley, Owens College.
Spencer, Charles Barré Phipps, Guy's Hospital.
Wilson, Frederick Wallace, Guy's Hospital.

PHYSIOLOGY ONLY.

SECOND DIVISION.

Bale, William Barker, Owens College.
Brockbank, Edward Mansfield, Owens College.
Ionides, Theodore Henry, University College.
Pearson, Reginald Spencer, Owens College and Manchester Royal Infirm.
Yeomans, James, University College.

### INSTRUCTIONS RESPECTING THE MODE OF ENTRY FOR THE INTERMEDIATE SCIENCE AND B.Sc. EXAMINATIONS.

The Registrar gives notice that all Candidates will henceforth be required to comply with the following instructions:—

1. *Not less than Five Weeks* before the commencement of the Examination every Candidate must apply to the Registrar for a *Form of Entry*, which, together with all particulars respecting the arrangements for the Examination, will be sent to him immediately on receipt of the application.

2. This Form, duly filled up, must be returned to the Registrar *not less than Four Weeks* before the commencement of the Examination, and with it, *in the same cover*, must be sent (*a*) the Candidate's *Certificate of Good Conduct*, and (*b*) his *Fee* for the Examination.

No Candidate's name will be placed on the List of Candidates unless his *Form of Entry, Certificate*, and *Fee* shall have been received at the University *on or before the Fourth Monday* before the commencement of the Examination.

As soon as possible after the closing of the List, and not previously, each Candidate's Certificate and Fee will be acknowledged, his Certificate will be returned, and a Number, by which he is to be designated throughout the Examination, will be assigned to him.

### Scraps and Gleanings.

Last Sunday was kept as Hospital Sunday at Leeds.

Five nurses were trained last year in the Belfast Lying-in Hospital.

The Leeds Ambulance was called out over four hundred times last year.

Hunts County Hospital takes paying patients at a charge of 30s. a-week.

There are two hundred cases of measles at Rainham, and all schools are closed.

Oldham Infirmary issued a satisfactory report at the beginning of the month.

Mr. Munro Smith has been elected assistant surgeon of Bristol Royal Infirmary.

The Mansion House Fund for the relief of the famine sufferers in China exceeds £14,000.

A complaint reaches us from Newcastle that the Hospital Sunday Fund decreases yearly.

The Guardians of Coventry have accepted Messrs. Steane's plans for the new Union Infirmary.

The proposal to build an infectious hospital at Clitheroe is held to be in abeyance for six months.

The reported overcrowding of Hatton Asylum is to come before the Rugby Board of Guardians.

New Brighton Convalescent Home received 878 patients last year. The Home is economically managed.

The North-Western Fever Hospital is to be used as a convalescent home for Asylums Board fever cases.

The new Children's Convalescent Home at West Kirby has received 72 inmates since it was opened last July.

Aberdeen has secured the needful £30,000 for the extension of its hospital; 311 patients were treated last year.

The Samaritan Society connected with Durham County Hospital supplies district nurses to the poor of the town.

Professor Virchow has become an honorary member of the famous Berlin Medical Society for Internal Complaints.

The Kent and Canterbury Hospital has at last got a balance on the right side. May the balance continue and increase.

The meeting of governors at Northampton Infirmary on the occasion of the election of a matron on February 4 was the largest on record.

Ripon Cottage Hospital has a rather large expenditure to report for last year, but then, hot-water apparatus has been added and a mortuary built.

### Amusements and Relaxation.

### NEW PUZZLE PRIZE SCHEME.

SECOND QUARTERLY COMPETITION COMMENCED JANUARY 5th, ENDS MARCH 30th, 1889.

1. Prizes will be awarded for the best Original Puzzles in Prose or Verse relating to any of the Social, Political, or Philanthropical topics of the day. Competitors are requested to *strictly* observe this rule, or their contributions will be discredited.
2. Puzzles may take any form which the ingenuity of the competitors suggest. Answers must accompany contributions, and the source of obscure or ambiguous terms be given.
3. No competitor will be allowed to take more than one prize during the Quarter.
4. Eight Prizes of Standard Books will be given, the choice of the book to be left to the winner. First, Second, Third, Fourth, and Fifth Prizes for best Original Puzzles, £1 1s., 15s., 10s. 6d., 7s. 6d., and 5s. each.
5. First, Second, and Third Prize for Solutions of 15s., 10s. 6d., and 5s. each.

N.B.—All contributions must reach the office, 38, Old Jewry, E.C., not later than the *First Post* on *Thursday* in each week.

### PUZZLES FOR SOLUTION.

**Eighth Week.**

No. 17. Double Acrostic.

1. Of the place where I was born
   I must *this* for ever be.
2. On some sunny, quiet morn,
   Thus to you *appears* the sea.
3. May it never be your doom
   To sink so low as this.
4. Standing in another's room,
   He fulfils that other's office.
5. In the lives of old world heroes,
   This myth holds a foremost place.
6. Reconcile flight with repose;
   O'er these run with steady pace.

Read downwards, omitting *initials* and *finals*, the name of one of England's princely sons. Tinie.

No. 18. Diamond Puzzle.

1. Here a vowel in frequent use,
   Which sometimes leads to its abuse.
2. To stag, to horse, to buffalo,
   Resemblances this beast can show.
3. Performer in the soldiers' band,
   With the drummer boy he takes his stand.
4. Here a term of endearment behold,
   To the babe which the mother's arms enfold.
5. This is a common but tiresome ill,
   Produced when the body has taken a chill.
6. Within their hidden recesses deep
   How many useful things we keep!
7. Dear to the childish appetite,
   Which doth in lollipops delight.
8. An ending to an adjective,
   When, as a verb, the word doth live.
9. A vowel which points out but one;
   And now, you see, the puzzle's done. Patience.

**Marks for Original Puzzles.**

| Names. | No. of Puzzles sent in Feb. 14. | No. of Marks Feb. 14. | Totals. | Names. | No. of Puzzles sent in Feb. 14. | No. of Marks Feb. 14. | Totals. |
|---|---|---|---|---|---|---|---|
| Cotonopolis... | 2 | 5 | 31 | Prior........... | — | — | 9 |
| Tinie............ | 3 | 6 | 31 | Lightowlers . | 2 | 2 | 13 |
| Jenny Wren . | — | — | 4 | Twentieth ... | — | — | 3 |
| Patience ...... | 3 | 7 | 34 | Crocodile...... | 1 | 2 | 5 |
| Padre ......... | — | — | 3 | Nabob ........ | — | — | 2 |

**Answers.**

No. 12. Double Acrostic.

```

V    er     B

I   rawad   I

C   orsica  A

T    abo    R

O    de     R

R    amr    I

I ngoldstad T

A   mbriz   I

```

No. 13. Diamond Puzzle.

```
          C
        C O T
      H O M E S
    C O P P E R S
  N U R S E R I E S
C O M P E T I T I O N
  P H Y S I C I A N
    M E N T H O L
      S K I L L
        C O D
          N
```

**Marks for Solution of Puzzles.**

Total Marks.—Flora (1), 6; Mater, 3; Scrooge (1), 6; Patience 2; Lady Clara (0) 5; Ruffles 4; Lauriston, 0; Reldas (1), 2; Jenny Wren, 2; Condorcet, 1; Tinie, 2.

[*The Figures in parentheses are Marks for the week ending Feb. 14th.*]

For Conditions, see last week's The Hospital.

